# The impact of spectacle lenses for myopia control on visual functions

**DOI:** 10.1111/opo.12878

**Published:** 2021-09-16

**Authors:** Yi Gao, Ee Woon Lim, Adeline Yang, Björn Drobe, Mark A Bullimore

**Affiliations:** ^1^ Research & Development Vision Sciences AMERA, Essilor International Singapore Singapore; ^2^ College of Optometry University of Houston Houston Texas USA

**Keywords:** children, contrast sensitivity, motion perception, myopia, myopia control, visual acuity

## Abstract

**Purpose:**

Spectacle lenses containing multiple small peripheral elements have been developed for myopia control in children. It is important that their effect on vision be quantified by (i) fixation through the peripheral portion, thereby using foveal vision and (ii) by fixation through the central portion and presentation of peripheral targets.

**Methods:**

The above approaches were used in five studies to evaluate two novel spectacle lens designs: spectacle lenses with Highly Aspherical Lenslets (HAL) and Slightly Aspherical Lenslets (SAL). A single vision lens served as a control. Visually normal adults participated in each study. The first two studies had subjects fixate through the periphery of the lenses. High and low (10%) contrast visual acuity was measured with the Freiburg Vision Test and reading speed for high and low contrast words measured with a sentence generator. The other three studies assessed peripheral vision while subjects fixated through the central portion of the lens. Peripheral contrast sensitivity was measured using two cycles per degree drifting Gabor stimuli. Peripheral motion perception was further evaluated using random dot stimuli. Finally, attention was measured using an established test of useful field of view with three levels of complexity.

**Results:**

The periphery of the HAL lens significantly reduced low contrast visual acuity, but not high contrast visual acuity, while the effect of the SAL lens was not significant for either. Neither test lens affected reading speed for high contrast words, but the HAL lens significantly affected performance for low contrast words. Neither test lens affected peripheral motion perception or useful field of view.

**Conclusions:**

Low contrast visual acuity and reading was slightly reduced while high contrast visual acuity was unaffected when fixating through the periphery of the novel lens designs. None of the peripheral measures of vision was affected by the novel lens designs.

**Key Points**

- This study comprehensively assessed the impact on various visual functions of two novel designs of myopia control spectacle lenses with aspherical lenslets.
- High contrast visual acuity and all the peripheral measures were unaffected by the novel lens designs while low contrast visual acuity through the periphery was slightly reduced.
- The two novel lens designs showed no clinically significant impact on tested central and peripheral visual functions; thus are safe to use for myopia control

## INTRODUCTION

The 21^st^ century has seen our perception of myopia transition from a benign refractive condition to a disease that represents an important public health issue. The American Academy of Ophthalmology's Board of Trustees, for example, believes that “myopia is a high‐priority cause of visual impairment, warranting a timely evaluation and synthesis of the scientific literature and formulation of an action plan to address the issue from different perspectives.”^1^ Increasing levels of myopia are strongly associated with an increased risk of a number of eye diseases including myopic maculopathy, open angle glaucoma, posterior subcapsular cataract and retinal detachment.^2^, ^3^, ^4^ Likewise, each additional dioptre of myopia is associated with a 30% increased risk of visual impairment.^4^

The heighted awareness of the consequences of myopia has led to a proliferation of drugs and devices to slow its progression.^2^ Overnight orthokeratology and dual‐focus soft contact lenses both slow progression by clinically meaningful amounts.^5^, ^6^ Atropine at high concentrations is probably the most effective of modalities,^3^, ^7^ but post‐treatment acceleration remains a concern,^8^ and the accompanying mydriasis and cycloplegia require optical management. Lower concentrations of atropine, principally 0.01% have been widely embraced,^9^ but recent clinical trials suggest that its one‐year efficacy is less than 0.25 D or 0.1 mm,^10^, ^11^, ^12^, ^13^ making it inferior to contemporary optical therapies.

Attempts at spectacle‐based myopia control date back to the middle of the last century.^14^ Progressive addition lenses (PALs) have limited efficacy, slowing progression by less than 0.25 D over two to three years^15^, ^16^ even in patients selected on the basis of near esophoria and accommodative lag.^17^ A 3‐year clinical trial found a 0.75 D and 0.25 mm slowing of progression with executive bifocals.^18^ It should be noted, however, that a previous, similar study found no effect.^19^

Newer spectacle lens designs have had similar mixed results. Concentric PALs designed to reduce peripheral hyperopic defocus have little or no benefit.^20^, ^21^ Greater success has been reported with lenses containing multiple small peripheral elements with positive power. Among children completing a 2‐year study, a lens with defocus incorporated multiple segments (DIMS) slowed myopic progression by around 0.4 D and axial elongation around 0.3 mm.^22^ One‐year results from a large randomised clinical trial demonstrated 0.53 D and 0.33 D slowing of myopia progression, and 0.23 mm and 0.11 mm slowing of axial elongation with spectacle lenses containing Highly Aspherical Lenslets (HAL) and Slightly Aspherical Lenslets (SAL), respectively.^23^ Both lenses consist of the same configuration of 11 concentric rings of aspherical lenslets centred on a 9 mm‐diameter clear central zone. Detailed description of the two lenses have been provided elsewhere (see *Figure* *1* in Bao *et al*.^23^). After 2 years, the HAL and SAL lenses slowed myopia progression by 0.80 and 0.42 D, and axial elongation by 0.35 and 0.18 mm, respectively.^24^

**FIGURE 1:**
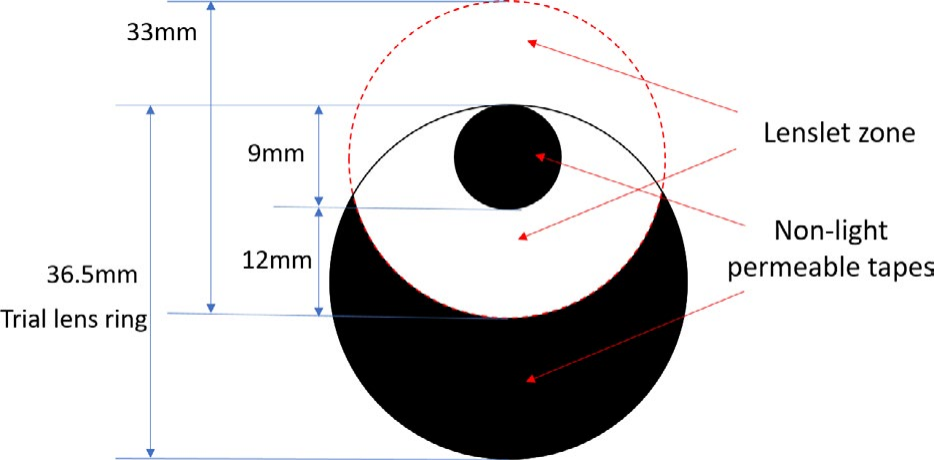
Test lenses that have been edged with the clear zone decentred and masked for assessing foveal vision through the peripheral area

If large numbers of children are to be prescribed these and other myopia control devices, it is important that vision with them be quantified. High contrast visual acuity is unaffected by dual‐focus soft contact lenses or overnight orthokeratology,^5^, ^25^, ^26^ but low contrast visual acuity measured with dilated pupils or at low luminance to promote larger pupils reveals small but potentially meaningful deficits.^25^, ^26^

While soft lenses move with changes in gaze, spectacle lenses do not, so the patient samples different areas of the lens—a desirable feature for bifocals and PALs. The influence of novel lens designs with peripheral elements on vision can thus be evaluated in two ways:

1. Having subjects fixate through the peripheral portion of the lens, thereby using their foveal vision.
2. Having subjects fixate through the central portion of the lens and present peripheral targets, thereby using their peripheral vision.

The first approach gives a measure of the limits placed on resolution due to the lens design and the visual acuity achieved should a patient turn their eyes to fixate a target through the peripheral portion of the lens. The second method assesses the impact of the lens design on peripheral vision. By measuring sensitivity to stimuli that are low contrast, moving or both, the effect of the lens design on hazard detection in peripheral vision may be evaluated. Both approaches have been used in a series of five studies described below to evaluate two novel spectacle lens designs.

## METHODS

### Lenses evaluated

Two novel lens designs, each containing peripheral lenslets, were evaluated in each study:

- HAL: Spectacle Lenses with Highly Aspherical Lenslets;
- SAL: Spectacle Lenses with Slightly Aspherical Lenslets.

Both lenses consist of the same configuration of 11 concentric rings of aspherical lenslets centred on a 9 mm‐diameter clear central zone. Detailed description of the two lenses have been provided elsewhere.^23^ A conventional single vision lens was used as a control. All lenses were polycarbonate. When foveal vision through the lens periphery was evaluated, the central zone was patched with tape in order to force the subjects to look through the zones containing the micro‐lenses (*Figure* *1*). The single vision lens (SVL) was also patched in the same way.

### Subjects

Essilor International Institutional Review Board approval was obtained for all studies and written informed consent obtained from all participants. Eight to 10 visually normal adults from a pool of 28 individuals participated in each study. Their age ranged from 19 to 47 years (mean = 28.9 ± 8.6 years) and their refractive error from −8.50 to +1.75 D spherical equivalent. Half of these individuals participated in more than one study, with one participating in all five. Myopes accounted for between 50 and 80% of participants in each study. All subjects were free from ocular disease, had visual acuity better than 0.1 logMAR (6/7.5) at the time of testing, and wore their habitual correction. The subject's dominant eye was tested with the other eye patched. Subjects were masked to lens type for all tests although sometimes subjects noticed that the lenses with lenslets looked different or caused subjective changes in vision when looking through the lenslets.

### Statistical analysis

Parametric methods were used to determine means and standard deviations. To evaluate differences in vision among the lenses, a repeated‐measures factorial analysis of variance (RMF‐ANOVA) was used, either with a single factor (lens) or with a second (e.g., contrast). When appropriate, post‐hoc Fisher's Protected Least Significant Difference (PLSD) tests were used to compare differences between pairs of lenses.

## FOVEAL VISUAL ACUITY THROUGH THE LENS PERIPHERY

### Stimuli

Visual acuity was evaluated using the Freiburg Vision Test (FrACT)^27^, ^28^ (michaelbach.de/fract.html). To estimate the acuity threshold, a best PEST (best Parameter Estimation by Sequential Testing) procedure is used in which a psychometric function having a constant slope on a logarithmic acuity scale is assumed. Measurement terminates after a fixed number of trials. The advantage of this test is that it is computerised and automated, and therefore limits examiner bias. A single letter allows fixation at one point, instead of fixating from top to bottom as required when using a regular visual acuity chart. The stimuli were presented on a gamma‐calibrated 22‐inch HP P1230 high‐resolution CRT monitor (HP, hp.com). Visual acuity was measured for high (100%) and low (10%) contrast (Weber). Screen luminance was 120 cd/m^2^.

### Procedure

Viewing distance was 3 m and room illumination was 10 lux. Ten participants attended two sessions on two separate days: one day for 100% optotype contrast and another for 10% optotype contrast. Two practice trials were implemented to minimise the effect of familiarity. In each session, subjects were tested with the three lenses in random order. Each condition was measured three times. The total duration of each session was around 45 min. Subjects indicated the orientation of the Landolt C using a keypad. Auditory feedback informed subjects whether their response was correct or incorrect. At the end of each trial, subjects' responses were converted into logMAR.

### Results

**FIGURE 2:**
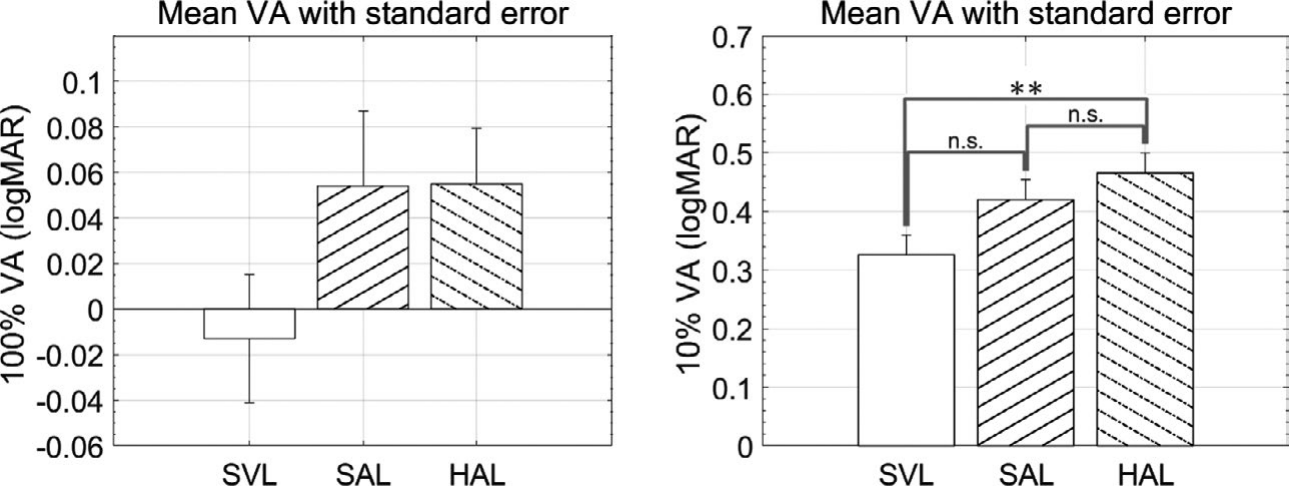
Visual acuity (VA) through three lenses at 100% (left) and 10% (right) contrast. Error bars represent standard errors. Asterisks signify significant difference between lenses. SVL, Single vision lens, SAL, Spectacle lens with slightly aspherical lenslets, HAL, Spectacle lens with highly aspherical lenslets

Mean visual acuity through the periphery of each lens is shown in *Figure* *2*. The asterisks signify a significant difference between pairs of lenses. High contrast visual acuity was not significantly affected by lens design (*F*_2,27_ = 1.83, *p* = 0.18), but low contrast visual acuity was affected (*F*_2,27_ = 4.31, *p* = 0.02). Post‐hoc testing showed that low contrast visual acuity with HAL was poorer than with the single vision lens. For the HAL design, high contrast visual acuity was reduced by 0.07 ± 0.029 logMAR (a little over half a line) and low contrast visual acuity was reduced by 0.14 ± 0.053 logMAR (around 1.5 lines). For the SAL design, high contrast visual acuity was reduced by 0.07 ± 0.047 logMAR (a little over half a line) and low contrast visual acuity was reduced by 0.10 ± 0.062 logMAR (one line). There was no significant difference in either high or low contrast acuity between HAL and SAL.

## FOVEAL READING SPEED THROUGH THE LENS PERIPHERY

### Stimuli

The reading task used an automatic sentence generator that measures maximum reading speed in adults,^29^ which was based and improved upon the generator described by Crossland *et al*.^30^ This in‐house sentence generator gives reading speeds that agree well (7% slower) with those obtained with the MNREAD test in adult readers.^31^ The generator can produce a large number of English sentences, which vary the length of four to seven words per sentence. The words are made up of nouns and verbs, with a variable length of one to 12 letters. An advantage of the sentence generator is that it eliminates the complex comprehension process that might alter the reading pattern depending on the difficulty.^32^ Sentences were displayed on a gamma‐calibrated digital 5‐inch screen (Blackmagic Video Assist, Blackmagic Design, blackmagicdesign.com) with a resolution of 1920 × 1080. The displaying software was written in Python 2.7.6 (Python, python.org) with the module Pygame 1.9.1 (Pygame, Pygame.org). English words were displayed on a white background, with a Weber contrast of 100% or 10%.

### Procedure

**FIGURE 3:**
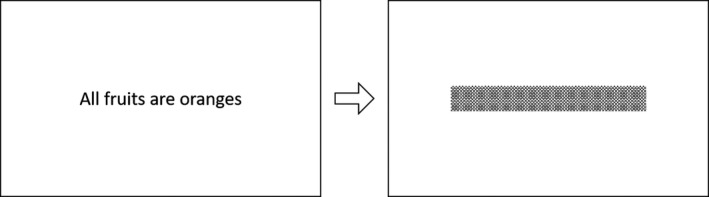
Sentence displayed on the screen (left), followed by a mask (right)

Viewing distance was 40 cm and room illumination was 10 lux. The font size was 52 pixels, which, at this viewing distance, corresponds to approximately 0.5 logMAR. Two modalities are possible: either the subject had to recite the whole sentence (oral recitation), or indicate whether the content was true or false (comprehension). In this study, reading speed was evaluated through oral recitation only in 10 participants. Each participant was given a demonstration of the task, followed by a familiarisation phase (with three repetitions). For a single trial, one sentence was displayed on the screen. The sentence disappeared and was replaced by a mask (*Figure* *3*). The subject would recite all the requested words orally. The time available to recite the sentence was unlimited. The investigator recorded whether the response was correct or not on the keyboard. The subject, when ready, pressed a key to display the next sentence. Reading speed was specified in display duration per word (in milliseconds). Therefore, if there were five words in the sentence and the display duration was 1 second, the display duration per word was 200 ms. A staircase method was used with the minimum display duration per word, which allowed the subject to read the whole sentence, determined as the mean of the last six reversals of the staircase, correctly.

Subjects were tested with the three lenses and two contrasts (100% vs. 10%) in random order. Subjects were masked to the type of lens. Each condition was measured three times and averaged. The total duration of the single session was around one hour.

### Results

**FIGURE 4:**
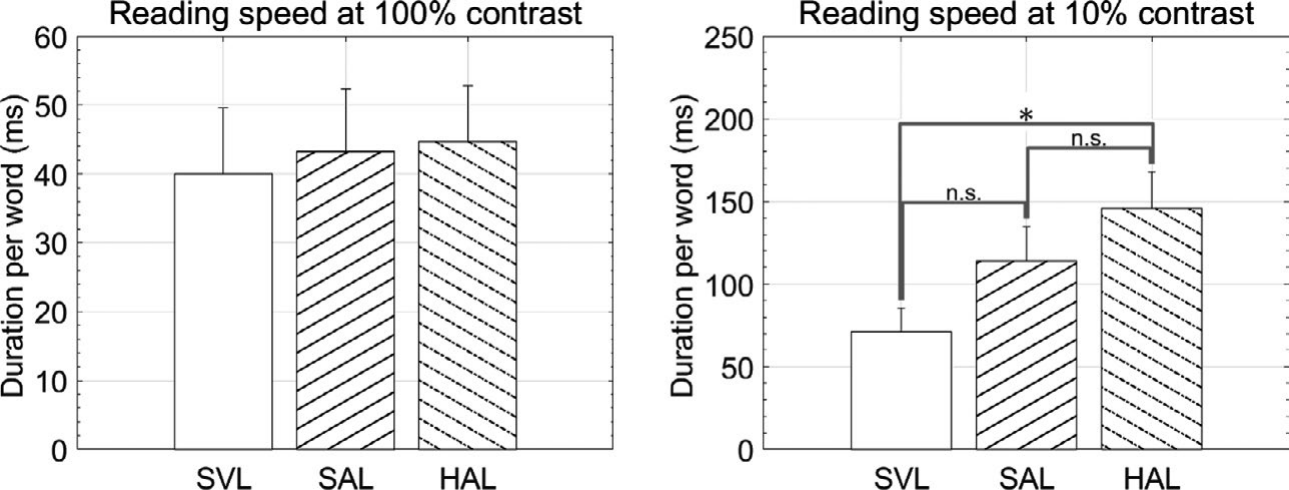
Threshold exposure duration for the three lenses for 100% (left) and 10% contrast (right), respectively. Error bars represent standard errors. *p < 0.05. SVL, Single vision lens, SAL, Spectacle lens with slightly aspherical lenslets, HAL, Spectacle lens with highly aspherical lenslets

Mean threshold duration per word through the periphery of each lens is shown in *Figure* *4*. At 100% contrast, lens type had no significant effect on threshold duration per word (*F*_2,27_ = 0.07, *p* = 0.93). At 10% contrast, thresholds were higher, but lens type had a significant effect on threshold duration per word (*F*_2,27_ = 3.7, *p* = 0.04). Post‐hoc testing showed that duration per word was shorter for the single vision lens than for the HAL lens. Not surprisingly, given the different findings at high and low contrast, a repeated measures two‐way ANOVA (three lenses and two contrasts) indicated that the interaction between lens and contrast approached statistical significance (*F*_2,54_ = 2.7, *p* = 0.08).

## PERIPHERAL MOTION DETECTION—GABOR PATCHES

### Stimuli

**FIGURE 5:**
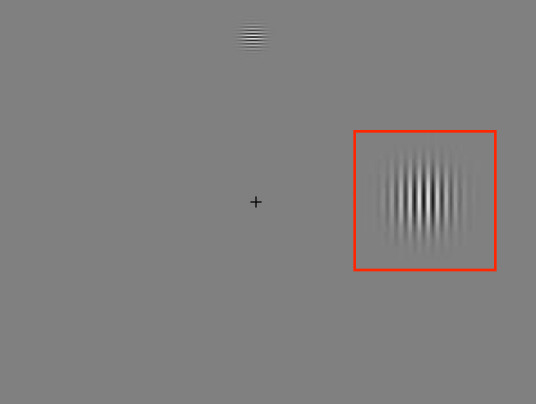
The phase‐shifting Gabor stimulus and its up location. An enlarged version of the stimulus is shown on the right for illustrative purposes

The stimulus was a phase‐shifting Gabor patch—a drifting grating within a fixed 2‐dimensional spatial Gaussian window. The grating had a spatial frequency of 2 cycles per degree and a speed of 4 degrees per second. The Gaussian mask had a diameter of 4 degrees and a sigma of 3 cycles. It is shown at one of four locations—left, right, up and down—at 26 degrees of eccentricity in the visual field (*Figure* *5*). At the four locations, the orientation of the grating was vertical for left and right, and horizontal for up and down. The presentation duration was 600 ms, and the inter‐trial interval was 500 ms. During stimulus onset and offset, the contrast of the stimulus gradually increased to the peak and decreased to zero following a sine and cosine temporal window, respectively, over 100 ms.

A cross as a fixation target was presented at the centre of the screen at all times. The task was to fixate the cross while attending to the periphery and to identify the location of the stimulus. The stimuli were presented on a gamma‐calibrated 43‐inch screen (model UA43MU6100K, Samsung, Samsung.com) with a refresh rate of 60 Hz. The mean screen luminance was 37.5 cd/m^2^. The stimuli and experiment procedures were generated using MATLAB R2018a (MathWorks, mathworks.com) with Psychophysics Toolbox Version 3 (Psychtoolbox, psychtoolbox.org).^33^, ^34^, ^35^

### Procedure

Viewing distance was 42 cm and room illumination was 10 lux. A 4‐alternative forced choice paradigm was employed with subjects indicating the identified location of the peripheral target on a keypad. An independent 1‐up 2‐down staircase was conducted concurrently for each location. Contrast of the Gabor stimulus varied within each staircase, starting at 100%. Each staircase was terminated when the number of trials reached 70 or the number of reversals reached 10. Threshold was calculated as the mean contrast level of the last six reversals for each staircase. One practice run was conducted prior to data collection. Eight subjects, masked to the lens type, were tested with the three lenses in random order. Each condition was measured twice. The total duration of the session was around 1 h.

### Results

**FIGURE 6:**
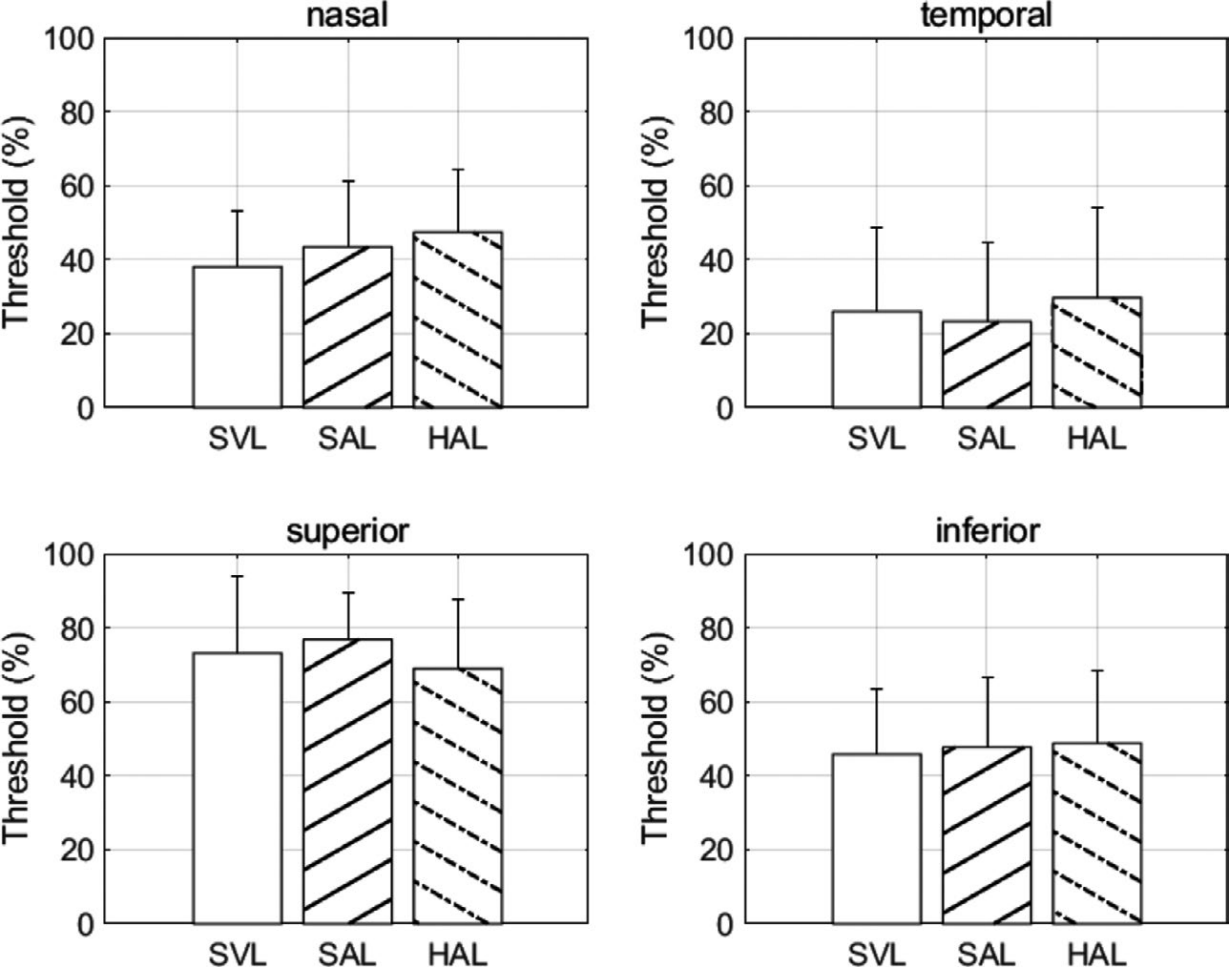
Contrast thresholds for peripheral motion at four peripheral locations through the three lenses. SVL, Single vision lens, SAL, Spectacle lens with slightly aspherical lenslets, HAL, Spectacle lens with highly aspherical lenslets

Contrast thresholds for each lens and each peripheral location are plotted in *Figure* *6*. There was no significant effect of lens at any location (*F*_2,27_ ≤ 0.71, *p* ≥ 0.50). In other words, the test lenses do not affect peripheral motion detection. Two‐way ANOVA showed a significant effect of location (*F*_3,108_ = 27.49, *p* < 0.0001).

## PERIPHERAL MOTION DETECTION—CARDINAL MOTION

### Stimuli

**FIGURE 7:**
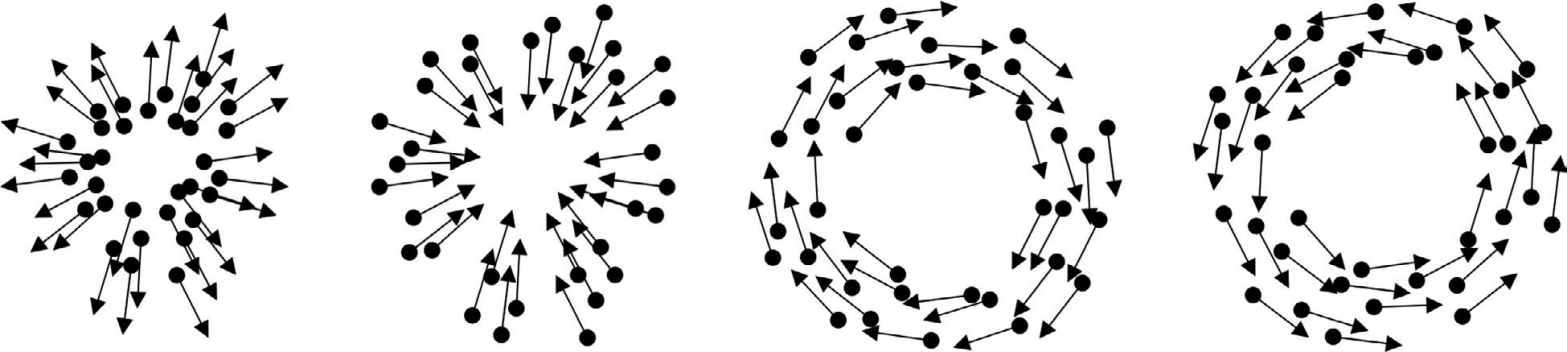
Cardinal motion directions tested. From left, expanding, contracting, clockwise and counter‐clockwise

**FIGURE 8:**
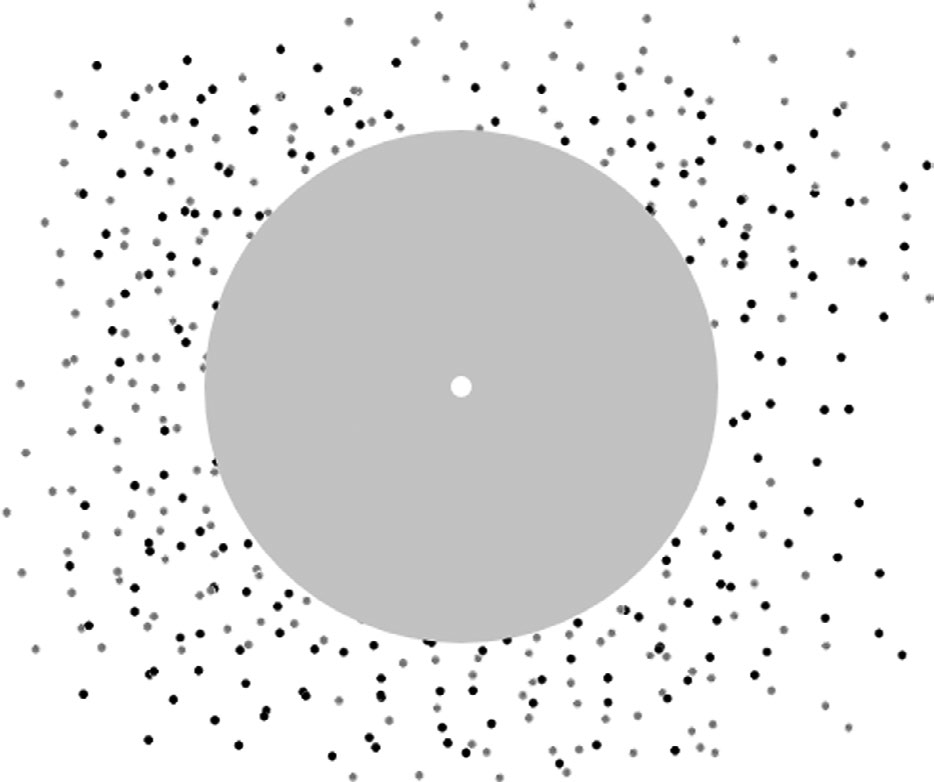
Example of the display with moving stimuli. The grey patch covered the central 18 degree diameter of the display while the central fixation cross remained visible

Coherent thresholds for global motion stimuli of four directions—expanding, contracting, clockwise and counter‐clockwise—were measured (*Figure* *7*). The stimuli comprised 360 randomly positioned dots that moved at 4 degrees per second with varying coherence. The motion is most easily defined as the frame‐wise increment in the polar coordinates of each randomly positioned dot, of which half were black and half were white. Dots were 6 min of arc in diameter and 100% contrast (Weber Contrast), displayed within a 40‐degree grey screen square with a mean luminance of 50 cd/m^2^ (*Figure* *8*). The central 18 degrees diameter of the display was masked, except for the central fixation. The motion had a ‘limited lifetime’ of two frames. After each dot moved, it was extinguished to be shown in a new random position. The frame‐rate of the motion display was 20 Hz (50 ms per position).

A white fixation circle at the centre of the screen was provided as a fixation target at all times. The task was to fixate the circle while attending to the periphery and identify the direction of the motion. Stimuli were presented on a gamma‐calibrated 43‐inch Samsung UA43MU6100K screen with a refresh rate of 60 Hz. The stimuli and experimental procedures were generated using MATLAB R2018a with Psychtoolbox 3.^33^, ^34^, ^35^ Viewing distance was 42 cm and room illumination was 10 lux.

### Procedure

Subjects were required to discriminate the direction of motion—expanding, contracting, clockwise and counter‐clockwise—in a one‐interval presentation (*Figure* *7*). There were 30 trials for each direction randomly displayed within a total of 120 trials for each lens. Percentage coherence motion thresholds were calculated—the lower the value, the higher the sensitivity. For each experiment, subjects looked through the middle of the clear zone of each lens. One practice run was conducted prior to data collection. Ten subjects were tested with two lenses in random order (SVL and HAL). To reduce subject burden, the SAL lens was not evaluated. Each condition was measured once. The total duration of the session was around 30 min.

### Results

**FIGURE 9:**
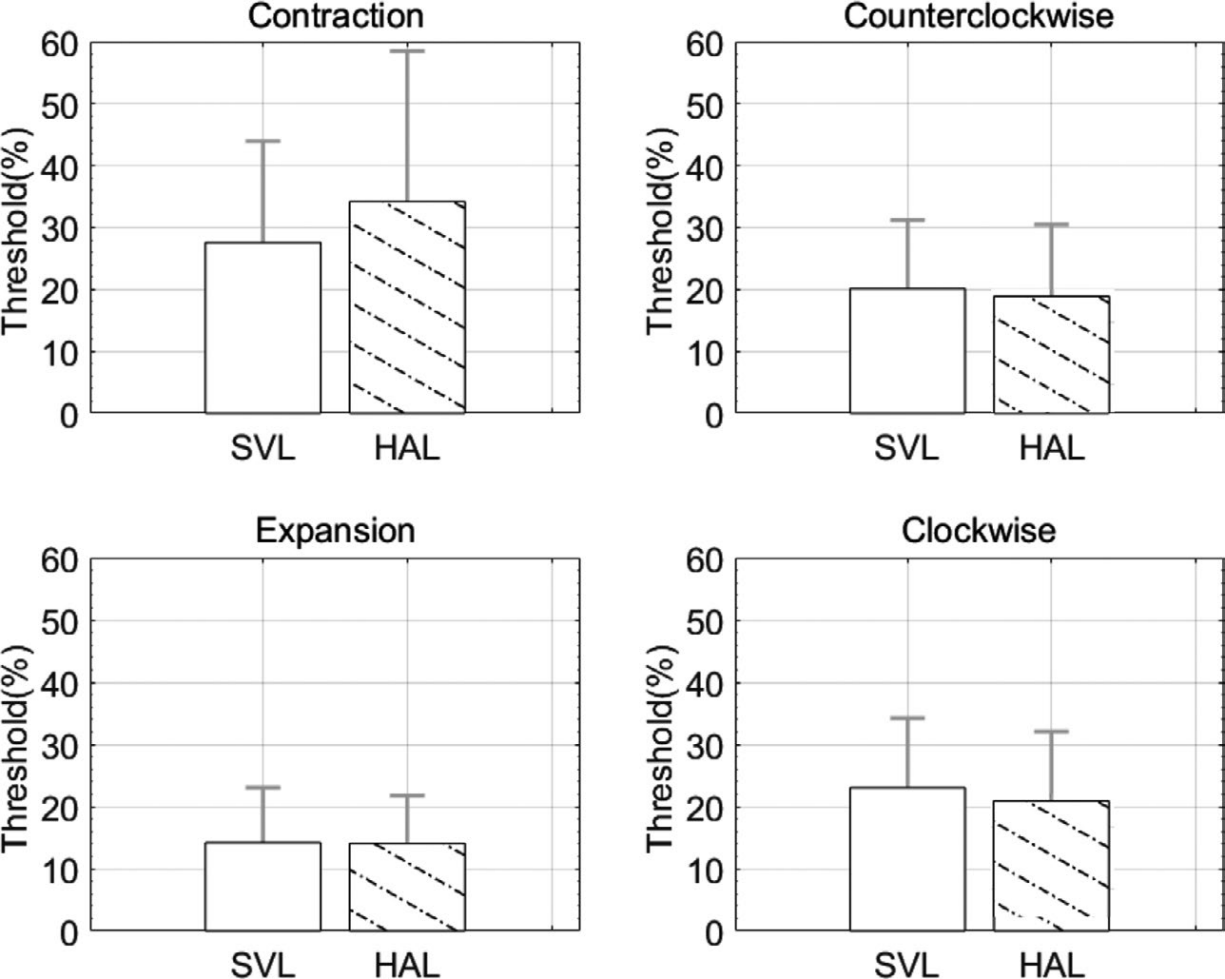
Coherence threshold for each lens and direction (error bars represent standard deviation). SVL, Single vision lens, HAL, Spectacle lens with highly aspherical lenslets

Coherence thresholds for each lens and direction are plotted in *Figure* *9*. There was no significant effect of lens on coherence threshold (*F*_1,72_ = 0.07, *p* = 0.80). In addition, there was no interaction between lens and the direction of motion (*F*_3,72_ = 0.41, *p* = 0.74).

## USEFUL FIELD OF VIEW

### Stimuli

The useful field of view is the visual area over which information can be extracted at a brief glance without eye or head movements.^36^, ^37^ It assesses visual attention and other aspects of visual processing. The useful field of view test consists of three subtests with increasing complexity:

1. Processing Speed: Determines a person's threshold for discriminating stimuli presented in central vision.
2. Divided Attention: Same as Subtest 1 but with the addition of a concurrent peripheral target location task.
3. Selective Attention: Same as Subtest 2 but with the addition of distracters.

The variable for all three is stimulus duration.

*Subtest 1*

**FIGURE 10:**
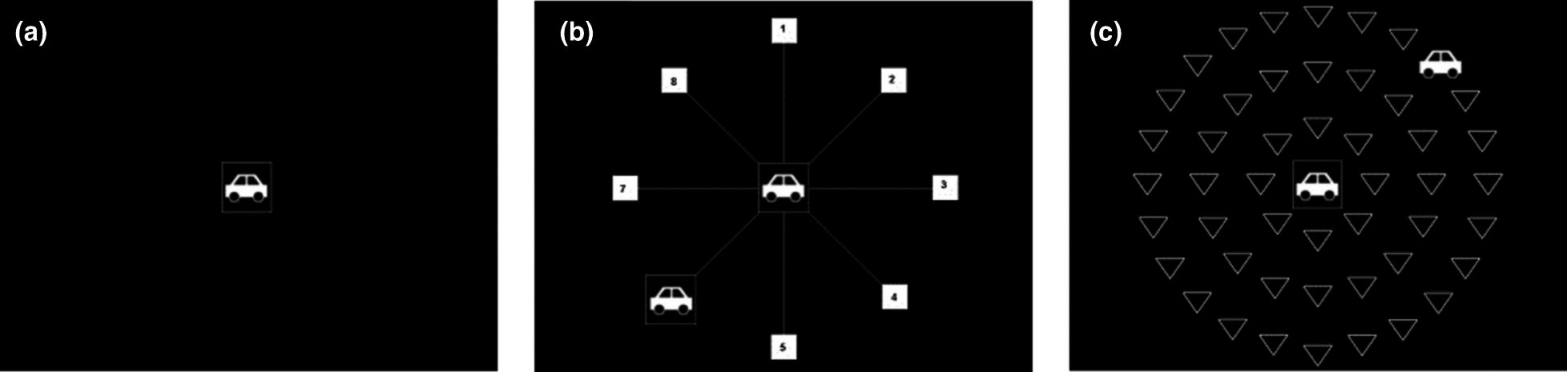
(a) Car stimulus presented on the screen during Subtest 1. (b) Two stimuli presented on the screen during Subtest 2. (c) Two stimuli with distractors presented on the screen during Subtest 3

The stimulus was a car or truck in the centre of the screen (*Figure* *10a*). The participant was required to identify the target presented.

*Subtest 2*

Two stimuli (one at the centre and one in the periphery) were presented on the screen at the same time (*Figure* *10b*). The participant was required to identify the target object in the centre and the location of target display in the periphery.

*Subtest 3*

This task was identical to Subtest 2, except that the target displayed in the periphery was embedded in distractors (*Figure* *10c*).

The stimuli were presented on a gamma‐calibrated 24‐inch calibrated Dell LCD screen (Dell, dell.com).

### Procedure

Useful field of view assessment was performed using commercially available software (Useful Field of View (UFOV) Assessment, BrainHQ, Posit Science, brainhq.com). Viewing distance was 40 cm and room illumination was <5 lux. The screen background luminance was dark (<1 cd/m^2^). At 40 cm, the eccentricity of the peripheral targets was 17 degrees. Eight participants attended a single session. In the beginning of the session, one practice run was conducted prior to data collection. Subjects were tested with the three lenses in random order. Each condition was measured twice. The total duration of each session was around 45 min.

Subjects reported their responses through mouse clicks identifying the object in the centre, the location of the peripheral stimulus or both. A two‐step size staircase algorithm was used to estimate thresholds, where “step” refers to display time. The algorithm started at 500 ms with a 50‐ms step size and reduced to 17 ms after the first incorrect response. The assessment ended early if there were three consecutive trials at the shortest (17 ms) or the longest (500 ms) presentation time.

### Results

**FIGURE 11:**
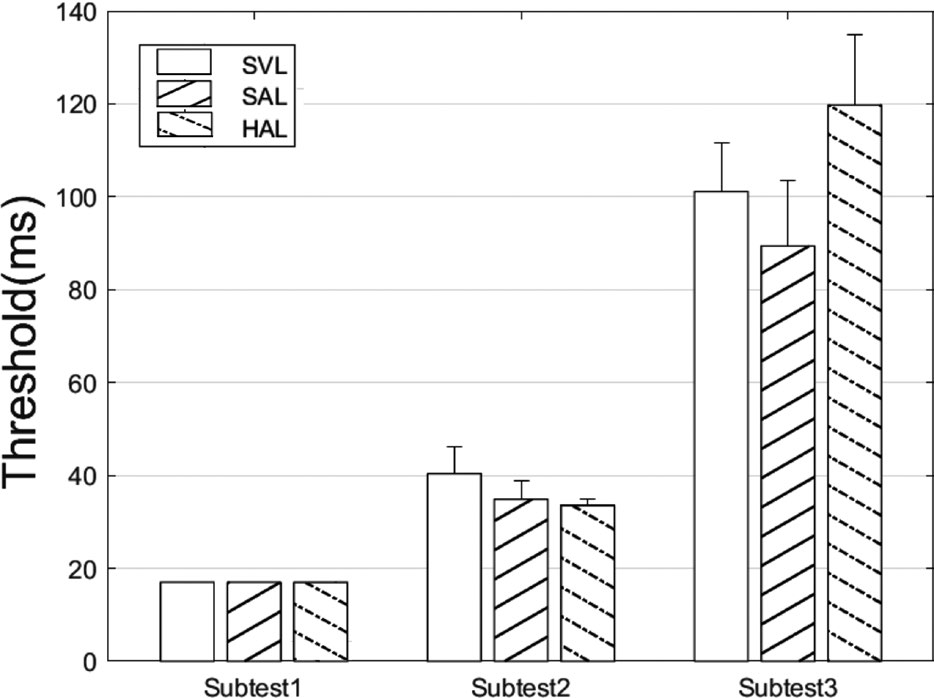
Threshold duration at 40 cm for three subtests and three lenses (error bars represent one standard deviation). SVL, Single vision lens, SAL, Spectacle lens with slightly aspherical lenslets, HAL, Spectacle lens with highly aspherical lenslets

Threshold presentation time for each lens and each subtest are plotted for the 40 cm viewing distance in *Figure* *11*. As expected, thresholds were lowest for Subtest 1 and highest for Subtest 3. For Subtest 1, all subjects could perform the task at the shortest duration of 17 ms, so no analysis is presented. There was no significant effect of lens for either of the other two subtests (Subtest 2: *F*_2,21_ = 0.76, *p* = 0.48; Subtest 3: *F*_3,28_ = 1.31, *p* = 0.29).

## DISCUSSION

The comprehensive studies described above quantify the effect of two experimental lens designs on visual functions. The Essilor^®^ Stellest™ lens (Essilor, essilor.com) is based on the same optical design as the HAL prototype, while the SAL design has not been commercialised to date. Therefore, the results for the SAL lens are discussed in less detail.

The first two studies examined vision when the subject fixated through the periphery of the lens containing the multiple lenslets. The HAL lens reduced high contrast visual acuity by 0.07 logMAR—just over half a line on a logMAR chart. The same lens reduced low contrast (10%) visual acuity by 0.14 logMAR (around 1.5 lines). In a separate study, high contrast visual acuity was measured in 50 myopic children using the same methodology and lenses.^38^ The HAL lens reduced visual acuity by 0.07 logMAR. In other words, the effect of the lens designs on visual acuity is the same in children and adults.

The HAL lens had no influence on reading performance for high contrast words, but significantly impacted performance for low contrast words. This finding is directly attributable to the size of the words relative to threshold visual acuity. It is well established that the reading rate increases as size is increased above threshold, and reaches maximum levels at sizes two to three times threshold. The words were equivalent to around 0.50 logMAR, which is about three times the size for threshold visual acuity (~10^0.5^) for both lenses (*Figure* *2*), and thus the reading rates would be expected to be similar. Conversely, at 10% contrast, visual acuity was 0.32 and 0.46 logMAR for the single vision and HAL lenses, respectively (*Figure* *2*). These are much closer to the size used for the reading task and thus mean threshold duration per word is dramatically increased for all lenses (*Figure* *4*). For the HAL lens, the size of the words in the reading task (0.50 logMAR) is barely above threshold (0.46 logMAR) and hence performance is more dramatically affected (*Figure* *4*).

In summary, the effect of the peripheral portion of the HAL lens on foveal vision was quantified and produced results that are consistent across the two studies. The lens produced a small reduction in high contrast visual acuity that does not affect reading performance for high contrast words. Low contrast visual acuity is affected to a slightly greater degree. Thus, reading performance for low contrast text is reduced because the text size used was very close to the threshold acuity under low contrast.

The results are generally better than recent reports of another spectacle lens with small peripheral segments.^39^ Near high contrast visual acuity through four peripheral areas containing the segments was significantly reduced by between 0.07 and 0.15 logMAR in adults; more severe than the non‐significant reduction in high contrast visual acuity loss of 0.07 logMAR in HAL and SAL. This result is consistent with that of another separate study in which better high contrast visual acuity in children was also found for HAL and SAL than the DIMS lens.^38^ Another recent investigation combined high sampling density optical metrology with pupil‐ and image‐plane numerical analyses to evaluate the same lens.^40^ Both visual inspection and wavefront metrology revealed that the lenslets gave a coverage factor of approximately 40%. In other words, there are multiple areas with single vision correction between the lenslets. Because of this, point spread functions were small, with sufficient image quality to view a 0.00 logMAR (6/6) letter.^40^

A further three experiments quantified the effect of the various lens designs on peripheral vision. Peripheral visual acuity was not measured, but the results can be predicted. Visual acuity is best at the fovea and decreases monotonically and rapidly as retinal eccentricity increases. The rate of decline is highly dependent on the stimulus used. For example, at 10 degrees, visual acuity for crowded letters, like those used in the first study, is 0.85 logMAR (6/42 or 4.2 cycles per degree) but 0.60 logMAR (6/24 or 7.5 cycles per degree) for single letters.^41^, ^42^ For grating stimuli, visual acuity improves further, but still only around 0.50 logMAR (6/19 or 9.5 cycles per degree).^43^ In other words, there is a difference in visual acuity of more than 0.30 logMAR between crowded letters and grating stimuli at 10 degrees. At 20 degrees, visual acuity for gratings (high frequency cut‐off) falls to 6 cycles per degree (6/30 or 0.70 logMAR)^44^ and to 1.00 logMAR (6/60 or 3 cycles per degree) for single letters.^42^ Thus, for crowded letters, visual acuity at 20 degrees eccentricity would be worse than 6/60 and the HAL lens would likely have no effect.

Lens design had no effect on peripheral motion perception (*Figure* *6*). Peripheral contrast sensitivity was measured using 2 cycles per degree stimuli, drifting at 4 degrees per second—equivalent to a temporal frequency of 8 Hz. At 20 degrees, peak contrast sensitivity is between 1 and 2 cycles per degree.^44^ Thus, the stimuli were close to the peak of the contrast sensitivity function. Contrast sensitivity at 10 degrees is similar for stationary and drifting gratings up to 10 Hz at 2 cycles per degree and higher.^43^ In other words, the results for moving stimuli may be extrapolated to contrast sensitivity for stationary targets. Like previous studies, contrast sensitivity was poorer in the vertical meridian than the horizontal meridian, particularly superiorly.^44^ Other researchers have demonstrated that contrast sensitivity is higher in the temporal field than the nasal field,^45^ consistent with the above results.

Peripheral motion perception was further evaluated using random dot stimuli (*Figure* *9*). Consistent with previous studies, sensitivity to expanding (centrifugal) motion was higher than that for contracting (centripetal) motion.^46^ Again, lens design had no significant effect on thresholds. Since there are no current plans to commercialise the SAL lens, it was excluded from the random dot study, but it would have no significant effect on peripheral motion detection because its lenslets are less aspherical than those on the HAL lens. Finally, a series of useful field of view experiments were conducted. As expected, thresholds increased with task complexity, but thresholds were not influenced by lens design (*Figure* *11*).

In summary, a series of experiments have quantified the modest reduction in foveal vision when a subject looks through the peripheral portion of the various lenses. When the subject looks through the central conventional portion of the lens, peripheral vision is unaffected. These studies represent the most comprehensive evaluation of the visual ramifications of myopia management technology. We are unaware of any similar measures made in subjects wearing dual‐focus soft contact lenses approved by the US Food and Drug Administration (FDA) for myopia control.^5^

It is interesting to analyse the possible interaction between myopia control lenses and normal visual development. Children who develop myopia are usually well beyond their critical period. Daw summarised that there are three periods in the development of visual acuity,^47^ with evidence primarily from studies of humans:

1. During the first 3 to 5 years of life, visual acuity develops from less than 6/60 to 6/6. During these years, acuity can be reduced by the various forms of deprivation leading to amblyopia.
2. Amblyopia is not confined to the first 3 to 5 years of life but can result from strabismus or anisometropia at any age, from several months to 7 years of age.
3. Recovery of acuity lost to amblyopia can occur in even older individuals. Eye care professionals have obtained positive results after sustained treatment of teenagers, and in a few cases adults who are affected by amblyopia.

In summary, given that the experimental lens designs evaluated had no measurable influence on peripheral visual function and that most myopia develops after the critical period of visual development,^48^ the lenses are highly unlikely to influence visual information processing or its development.

### Comparisons with multifocals

It is important to contrast the properties of these experimental lenses with conventional multifocal spectacle lenses. Considering first a progressive addition lens (PAL), there is an area of increased positive power in the lower field that will reduce distance visual acuity. If a patient looks through this region, their distance vision will be reduced to a greater extent than the above experimental lens designs. Likewise, but often forgotten, there is unwanted oblique astigmatism in the horizontal meridian of all PALs that increases with greater distance from the vertex line.^49^ This astigmatism is a necessary consequence of the power progression and the maximum level of astigmatism will be similar in magnitude to the add power.^49^, ^50^ Thus, a patient wearing a PAL with a +2 D reading addition, who looks to the left or right will experience around 2 D of oblique astigmatic defocus. Again, their vision through the lens periphery would be expected to be reduced more than through the above experimental lens designs.^51^

Bifocal lenses have two distinct areas of different power. Thus, distance vision is consistent above and either side of the lens centre. Below the lens centre, the positive lens power will reduce distance visual acuity in a manner similar to a PAL. A further feature of a bifocal is that the abrupt change in power leads to a prismatic effect or “jump” at the top of the near segment. This can cause an apparent displacement of fixed objects. A 1‐year prospective study of 156 older adults (mean age 76.5 years) of whom 87 were multifocal wearers (76 of them bifocals) found that the multifocal wearers were more than twice as likely to fall than single vision wearers (odds ratio = 2.29, 95% CI = 1.06–4.92).^52^ Multifocal wearers were also more likely to fall because of a trip, when outside their home, and when walking up or down stairs. There is some evidence that providing patients with single vision distance spectacles and recommending they wear them for walking and outdoor activities can reduce falls.^53^

The experimental lens designs evaluated here contain no pronounced prismatic effects due to the small aperture of the lenslets and the repetitive design. Thus, problems with stairs are unlikely to occur. Furthermore, the lenses are intended to be worn by children who are at less risk of bone fractures when falling than older adults.^54^ This younger population have yet to reach driving age and thus there are no other obvious safety concerns. In summary, there should be less risk of adverse events with these new lens designs compared to traditional multifocal lenses, some of which are advocated for myopia control in children.^16^, ^18^

### Summary

When fixating through the peripheral portion of the HAL and SAL lenses, high contrast visual acuity was not affected in adults; low contrast visual acuity was reduced only by the HAL lens. Reading speed was reduced by HAL only at low contrast, due to the size used being very close to low contrast visual acuity. None of the peripheral measures of vision including peripheral contrast detection, peripheral global motion processing and useful field of view, were affected by HAL or SAL. The series of measures in the current study presented a comprehensive evaluation of visual performance through the lenslets in both foveal and peripheral vision and indicated its safety in visual development for applying in the paediatric population for myopia control purposes.

## CONFLICT OF INTEREST

Yi Gao, Björn Drobe, Adeline Yang and Ee Woon Lim are employees of Essilor International. Mark Bullimore is a consultant for Alcon Research, Inc., Apellis, Inc., Arctic Vision, Inc., Ascpleix, Inc., CooperVision, Inc., Corneagen, Inc., Essilor International S.A., Essilor Instruments, Inc, Eyenovia, Inc., Genentech, Inc., Johnson & Johnson Vision, Inc., Novartis, AG, Presbia, Inc., Vyluma, Inc., and is the sole owner of Ridgevue Publishing, LLC, Ridgevue Technologies LLC, and Ridgevue Vision LLC. Preparation of this paper was supported by Essilor International.

## AUTHOR CONTRIBUTIONS

**Yi Gao:** Conceptualization (equal); Data curation (equal); Formal analysis (equal); Investigation (equal); Methodology (equal); Writing‐review & editing (equal). **Ee Woon Lim:** Data curation; Formal analysis; Investigation (equal); Methodology (equal); Writing‐review & editing (equal). **Adeline Yang:** Data curation; Formal analysis; Investigation (equal); Methodology (equal); Writing‐review & editing (equal). **Björn Drobe:** Conceptualization (equal); Data curation (equal); Methodology (equal); Supervision (equal); Writing‐review & editing. **Mark A. Bullimore:** Writing‐original draft (equal).
